# Serum 25-Hydroxyvitamin D, Calcium-Phosphate Profile, and Bone Mineral Density Across Normal, Osteopenic, and Osteoporotic Adults: A Hospital-Based Cross-Sectional Study

**DOI:** 10.7759/cureus.107775

**Published:** 2026-04-26

**Authors:** Veeracholan V, Sumanth Kumar B, Ponnazhagan K, Muninathan N

**Affiliations:** 1 Department of Biochemistry, Meenakshi Medical College Hospital and Research Institute, Meenakshi Academy of Higher Education and Research, Kanchipuram, IND; 2 Central Research Laboratory, Meenakshi Medical College Hospital and Research Institute, Meenakshi Academy of Higher Education and Research, Kanchipuram, IND

**Keywords:** 25-hydroxyvitamin d, bone mineral density, calcium, osteoporosis, phosphate, vitamin d

## Abstract

Background: This study evaluated the association of serum 25-hydroxyvitamin D (25(OH)D) with bone mineral density (BMD), skeletal status, serum calcium, and serum phosphate among adults undergoing bone health assessment.

Methods: This hospital-based cross-sectional study was conducted in the Department of Biochemistry at the Meenakshi Medical College Hospital and Research Institute, Kanchipuram, India, from January 2025 to December 2025. Ninety adult participants with complete biochemical and dual-energy X-ray absorptiometry (DXA) data were included. Adults aged 40 years or older with complete biochemical and DXA data were included. Participants were classified as vitamin D sufficient, insufficient, or deficient, with 30 individuals in each group. Skeletal status was categorized as normal, osteopenia, or osteoporosis using standard T-score criteria. Data were analyzed using chi-square and one-way analysis of variance.

Results: Normal BMD was observed more frequently in the vitamin D-sufficient group, whereas osteopenia and osteoporosis were more frequent in the vitamin D-insufficient and vitamin D-deficient groups (p < 0.001). Mean serum 25(OH)D, BMD, serum calcium, and serum phosphate were lower across osteopenia and osteoporosis categories than in the normal BMD category. Mean age did not differ significantly across skeletal categories.

Conclusions: In this selected hospital-based cross-sectional cohort, lower serum 25(OH)D levels were observed among participants with osteopenia and osteoporosis than those with normal BMD. These findings suggest that biochemical parameters may be interpreted alongside densitometric assessment, while recognizing the limitations of the cross-sectional design and lack of confounder adjustment.

## Introduction

Osteoporosis and osteopenia are common metabolic skeletal conditions characterized by reduced bone mineral density (BMD) and increased fracture risk. Their development is influenced by age, sex, hormonal status, nutritional status, physical activity, mineral balance, and comorbid disease. Among the biochemical factors related to skeletal health, vitamin D and calcium remain clinically important because of their established roles in bone mineralization, skeletal maintenance, and osteoporosis prevention [1-4].

Serum 25-hydroxyvitamin D (25(OH)D) is widely used as the principal circulating marker of vitamin D status. Vitamin D deficiency may reduce intestinal calcium absorption and may contribute to secondary disturbances in mineral metabolism. However, vitamin D does not act in isolation. Its skeletal relevance is linked to calcium availability, phosphate balance, parathyroid response, and broader nutritional factors that influence bone remodeling and mineralization [5-7].

Several observational studies have reported an association between lower serum 25(OH)D levels and reduced BMD or higher osteoporosis risk, particularly in postmenopausal women and selected endocrine populations [8-10]. However, the strength of this association is not uniform across all settings. Differences in population characteristics, skeletal sites assessed, assay methods, comorbid conditions, and adjustment for confounding variables may influence the observed relationship between vitamin D status and BMD.

Calcium and phosphate are also relevant in the assessment of skeletal metabolism because they reflect the mineral environment in which bone remodeling and mineralization occur. Altered calcium and phosphate levels have been reported among individuals with osteoporosis, and other micronutrients such as magnesium may also contribute to bone metabolism [11-14]. These observations support interpreting vitamin D status, in conjunction with mineral parameters, rather than as an isolated biochemical marker.

At the same time, published evidence includes studies showing weak or nonsignificant associations between serum 25(OH)D, routine biochemical parameters, and BMD, particularly in chronic disease populations or specific clinical subgroups [15-18]. Therefore, the relationship between vitamin D status and skeletal health should be interpreted with caution and in the context of study design, population characteristics, and unmeasured confounding factors.

Although the association between vitamin D status and skeletal health is well recognized, hospital-based data examining serum 25(OH)D, together with calcium, phosphate, and dual-energy X-ray absorptiometry (DXA)-defined skeletal categories, remain useful for describing local biochemical and densitometric patterns. The present study was therefore designed as a hospital-based cross-sectional assessment to evaluate the relationship between serum 25(OH)D and BMD, skeletal status, serum calcium, and serum phosphate among adults undergoing bone health evaluation. The study was intended to describe observed associations and not to establish causality or independent prediction.

## Materials and methods

This was a hospital-based analytical cross-sectional study conducted in the Department of Biochemistry, Meenakshi Medical College Hospital and Research Institute, Kanchipuram, India, from January 2025 to December 2025. The study was designed to evaluate the relationships among serum 25(OH)D, BMD, skeletal status, serum calcium, and serum phosphate in adults undergoing bone health evaluation.

As the study was cross-sectional and observational, the findings were interpreted as associations only. The study was not designed to establish causality, temporal sequence, or independent prediction. Ethical clearance was obtained from the Institutional Ethics Committee of Meenakshi Medical College Hospital and Research Institute (MMCH and RI IEC/PG/13/NOV/24).

Study population and sampling

Adults aged 40 years or older who underwent bone health evaluation during the study period and had complete data on serum 25(OH)D, serum calcium, serum phosphate, and BMD were considered eligible for inclusion. The study population was hospital-based and therefore may not represent the general community population.

A total of 90 eligible participants were included in the final analysis. Participants were categorized according to serum 25(OH)D concentration into vitamin D-sufficient, vitamin D-insufficient, and vitamin D-deficient groups, with 30 participants in each category. This equal group-wise distribution was used for comparative analysis across vitamin D strata. Therefore, the distribution of vitamin D status in this study should not be interpreted as a prevalence estimate for the source population.

No formal a priori sample size calculation was performed. The sample size was determined pragmatically based on the availability of eligible participants with complete biochemical and densitometric data during the study period. Accordingly, the study should be interpreted as exploratory and descriptive rather than confirmatory. The modest sample size and lack of formal power estimation were considered methodological limitations.

Inclusion and exclusion criteria

Participants were included if they were aged above 40 years, underwent bone health evaluation during the study period, and had available measurements of serum 25(OH)D, serum calcium, serum phosphate, and BMD by DXA.

Participants with conditions or treatments known to substantially influence bone metabolism or mineral homeostasis were excluded. These included chronic kidney disease, chronic liver disease, malabsorption syndromes, primary parathyroid disorders, malignancy, prolonged corticosteroid therapy, anticonvulsant use, and current or previous antiosteoporotic treatment.

Vitamin D classification

Serum 25(OH)D was used as the biochemical marker of vitamin D status. Participants were classified as vitamin D sufficient if serum 25(OH)D was ≥30 ng/mL, vitamin D insufficient if serum 25(OH)D was 20-29.9 ng/mL, and vitamin D deficient if serum 25(OH)D was <20 ng/mL. These categories were used for group-wise comparison with skeletal status and biochemical parameters.

Data collection and biochemical assessment

Demographic data, including age and sex, were recorded for all participants. Biochemical variables included serum 25(OH)D, serum calcium, and serum phosphate. These variables were selected because of their clinical relevance to vitamin D status, mineral balance, and skeletal metabolism.

Venous blood samples were collected under standard aseptic precautions and processed in the institutional biochemistry laboratory. Serum 25(OH)D was measured using an automated immunoassay-based method. Serum calcium and serum phosphate were measured using routine colorimetric biochemical methods. Serum 25(OH)D was expressed in nanograms per milliliter, while serum calcium and serum phosphate were expressed in milligrams per deciliter.

Detailed information regarding assay calibration, intra-assay and inter-assay variability, fasting status, and seasonal timing of vitamin D sampling was not available for analysis. These factors were therefore considered while interpreting the biochemical findings.

BMD assessment

BMD was assessed by DXA. Measurements were recorded in grams per square centimeter. Skeletal status was categorized according to standard T-score criteria. Normal BMD was defined as a T-score greater than or equal to -1.0. Osteopenia was defined as a T-score between -1.0 and -2.5. Osteoporosis was defined as a T-score less than or equal to -2.5. For methodological consistency, skeletal classification was based on standard densitometric interpretation at clinically relevant skeletal sites, including the lumbar spine and/or proximal femur, according to available DXA data.

Important regulators and potential confounders of bone metabolism, including parathyroid hormone, magnesium, bone turnover markers, body mass index, menopausal status, dietary calcium intake, sunlight exposure, physical activity, and vitamin D supplementation history, were not available in the dataset and were not included in the analysis. Therefore, residual confounding could not be excluded.

Study outcomes

The primary outcome was the distribution of DXA-defined skeletal status, categorized as normal BMD, osteopenia, and osteoporosis, across vitamin D categories. Secondary outcomes included the sex-wise distribution of skeletal status within each vitamin D category and comparison of mean serum 25(OH)D, BMD, age, serum calcium, and serum phosphate across skeletal-status categories. These outcomes were evaluated as cross-sectional associations only.

Statistical analysis

The association between sex and skeletal-status categories within each vitamin D group was assessed using the chi-square test. The overall association between the vitamin D category and skeletal status was also assessed using the chi-square test. Continuous variables, including serum 25(OH)D, BMD, age, serum calcium, and serum phosphate, were compared across skeletal-status categories using one-way analysis of variance. Because the available dataset did not include several important confounding variables, including parathyroid hormone, magnesium, menopausal status, body mass index, dietary intake, sunlight exposure, and physical activity, multivariable adjustment was not performed. Therefore, the statistical findings were interpreted as unadjusted associations. All statistical tests were two-tailed, and a p value of less than 0.05 was considered statistically significant. Given the exploratory nature of the study, p values were interpreted cautiously in relation to the study design, sample size, equal vitamin D group distribution, and absence of confounder adjustment.

## Results

In the vitamin D-sufficient group, 18 were female and 12 were male participants. Among female participants, 13/18 (72.2%) had normal BMD, 3/18 (16.7%) had osteopenia, and 2/18 (11.1%) had osteoporosis. Among male participants, 9/12 (75.0%) had normal BMD, 2/12 (16.7%) had osteopenia, and 1/12 (8.3%) had osteoporosis. Overall, 22/30 (73.3%) participants in this group had normal BMD. The sex-wise distribution of skeletal status was not statistically significant (p = 0.969) (Table 1).

**Table 1:** Distribution of participants with sufficient serum vitamin D concentration according to sex and bone mineral density Values are presented as n (%). The p value represents sex-wise comparison of skeletal-status distribution using the chi-square test

Sex	n	Normal, n (%)	Osteopenia, n (%)	Osteoporosis, n (%)	p value
Female	18	13 (72.2)	3 (16.7)	2 (11.1)	0.969
Male	12	9 (75.0)	2 (16.7)	1 (8.3)	
Total	30	22 (73.3)	5 (16.7)	3 (10.0)	

In the vitamin D-insufficient group, 19 were female and 11 were male participants. Among female participants, 3/19 (15.8%) had normal BMD, 9/19 (47.4%) had osteopenia, and 7/19 (36.8%) had osteoporosis. Among male participants, 2/11 (18.2%) had normal BMD, 6/11 (54.5%) had osteopenia, and 3/11 (27.3%) had osteoporosis. Overall, 5/30 (16.7%) participants had normal BMD, while 25/30 (83.3%) had either osteopenia or osteoporosis. The sex-wise distribution of skeletal status was not statistically significant (p = 0.866) (Table 2).

**Table 2:** Distribution of participants with insufficient serum vitamin D concentration according to sex and bone mineral density Values are presented as n (%). The p value represents sex-wise comparison of skeletal-status distribution using the chi-square test

Sex	n	Normal, n (%)	Osteopenia, n (%)	Osteoporosis, n (%)	p value
Female	19	3 (15.8)	9 (47.4)	7 (36.8)	0.866
Male	11	2 (18.2)	6 (54.5)	3 (27.3)	
Total	30	5 (16.7)	15 (50.0)	10 (33.3)	

In the vitamin D-deficient group, 19 were female and 11 were male participants. Among female participants, 3/19 (15.8%) had normal BMD, 9/19 (47.4%) had osteopenia, and 7/19 (36.8%) had osteoporosis. Among male participants, 1/11 (9.1%) had normal BMD, 6/11 (54.5%) had osteopenia, and 4/11 (36.4%) had osteoporosis. Overall, 4/30 (13.3%) participants had normal BMD, while 26/30 (86.7%) had either osteopenia or osteoporosis. The sex-wise distribution of skeletal status was not statistically significant (p = 0.858) (Table 3).

**Table 3:** Distribution of participants with deficient serum vitamin D concentration according to sex and bone mineral density Values are presented as n (%). The p value represents sex-wise comparison of skeletal-status distribution using the chi-square test

Sex	n	Normal, n (%)	Osteopenia, n (%)	Osteoporosis, n (%)	p value
Female	19	3 (15.8)	9 (47.4)	7 (36.8)	0.858
Male	11	1 (9.1)	6 (54.5)	4 (36.4)	
Total	30	4 (13.3)	15 (50.0)	11 (36.7)	

Overall distribution of skeletal status across vitamin D categories

When skeletal status was compared across vitamin D categories, normal BMD was observed in 22/30 (73.3%) participants in the vitamin D-sufficient group, 5/30 (16.7%) in the vitamin D-insufficient group, and 4/30 (13.3%) in the vitamin D-deficient group. Osteopenia and osteoporosis were more frequent in the insufficient and deficient groups than in the sufficient group. The association between vitamin D category and skeletal status was statistically significant (p < 0.001) (Table 4).

**Table 4:** Overall distribution of skeletal status across vitamin D categories Values are presented as n (%). The p value represents the comparison of skeletal-status distribution across vitamin D categories using the chi-square test

Vitamin D category	n	Normal, n (%)	Osteopenia, n (%)	Osteoporosis, n (%)	p value
Sufficient	30	22 (73.3)	5 (16.7)	3 (10.0)	<0.001
Insufficient	30	5 (16.7)	15 (50.0)	10 (33.3)	
Deficient	30	4 (13.3)	15 (50.0)	11 (36.7)	
Total	90	31 (34.4)	35 (38.9)	24 (26.7)	

Comparison of serum 25(OH)D, BMD, and age across skeletal categories

Participants were also analyzed according to skeletal category. Mean serum 25(OH)D was 38.62 ± 3.92 ng/mL in participants with normal BMD, 20.93 ± 2.60 ng/mL in those with osteopenia, and 10.85 ± 1.60 ng/mL in those with osteoporosis. Mean BMD was 0.982 ± 0.09 g/cm² in the normal BMD group, 0.844 ± 0.08 g/cm² in the osteopenia group, and 0.615 ± 0.06 g/cm² in the osteoporosis group. These differences were statistically significant for serum 25(OH)D and BMD. Mean age did not differ significantly across skeletal categories (p = 0.763) (Table 5).

**Table 5:** Comparison of serum 25-hydroxyvitamin D concentration, BMD, and age across skeletal categories Values are presented as mean ± standard deviation. p values were obtained using one-way analysis of variance BMD: bone mineral density

Variable	Normal BMD (n = 31)	Osteopenia (n = 35)	Osteoporosis (n = 24)	p value
Serum 25(OH)D (ng/mL)	38.62 ± 3.92	20.93 ± 2.60	10.85 ± 1.60	<0.001
BMD (g/cm²)	0.982 ± 0.09	0.844 ± 0.08	0.615 ± 0.06	<0.001
Age (years)	48.37 ± 4.92	49.23 ± 4.35	48.65 ± 4.55	0.763

Comparison of serum calcium and phosphate across skeletal categories

Serum calcium and phosphate also differed across skeletal categories. Mean serum calcium was 9.15 ± 0.42 mg/dL in participants with normal BMD, 8.65 ± 0.51 mg/dL in those with osteopenia, and 7.80 ± 0.63 mg/dL in those with osteoporosis. Mean serum phosphate was 3.54 ± 0.34 mg/dL in the normal BMD group, 3.01 ± 0.39 mg/dL in the osteopenia group, and 2.50 ± 0.46 mg/dL in the osteoporosis group. Both differences were statistically significant (Table 6).

**Table 6:** Serum calcium and serum phosphate levels across skeletal categories Values are presented as mean ± standard deviation. p values were obtained using one-way analysis of variance BMD: bone mineral density

Parameter	Normal BMD (n = 31)	Osteopenia (n = 35)	Osteoporosis (n = 24)	p value
Serum calcium (mg/dL)	9.15 ± 0.42	8.65 ± 0.51	7.80 ± 0.63	<0.001
Serum phosphate (mg/dL)	3.54 ± 0.34	3.01 ± 0.39	2.50 ± 0.46	<0.001

## Discussion

In this hospital-based cross-sectional study, serum 25(OH)D, BMD, serum calcium, and serum phosphate differed across DXA-defined skeletal categories. Normal BMD was more frequent in the vitamin D-sufficient group, whereas osteopenia and osteoporosis were more frequently observed among participants classified as vitamin D-insufficient or vitamin D-deficient. When participants were analyzed according to skeletal status, mean serum 25(OH)D, BMD, calcium, and phosphate were lower in the osteopenia and osteoporosis groups than in the normal BMD group. These observations suggest an association between vitamin D status, mineral profile, and skeletal category in this selected hospital-based cohort. However, because the study was cross-sectional and based on unadjusted comparisons, the findings should be interpreted as descriptive associations rather than evidence of causality or independent prediction.

The observed pattern is biologically plausible and is consistent with the established role of vitamin D and calcium in skeletal metabolism. Vitamin D contributes to calcium absorption and mineral homeostasis, while calcium availability remains essential for skeletal mineralization and maintenance [1-4]. Nutritional and metabolic factors may also influence bone health through several interacting pathways rather than through vitamin D alone [5-7]. In the present study, the lower mean 25(OH)D values among participants with osteopenia and osteoporosis are therefore directionally consistent with known physiology. Still, it would be inappropriate to infer from these data that low vitamin D was the direct cause of reduced BMD, because several important determinants of bone metabolism were not assessed.

The findings are also broadly aligned with some previous observational studies, although direct comparisons must be made with caution. Wang and Yang reported an association between serum 25(OH)D levels and the risk of osteoporosis in postmenopausal women [8]. Chen et al. also observed relationships between vitamin D status, BMD, bone turnover markers, and parathyroid hormone in Chinese postmenopausal women with osteopenia and osteoporosis [9]. Dhanwal et al. reported adverse skeletal findings in vitamin D-deficient patients with hyperthyroidism, representing a selected endocrine disease setting [10]. These studies support the relevance of vitamin D in bone health assessment, but their populations differ from the present hospital-based adult cohort. Therefore, they should be considered supportive background evidence rather than direct confirmation of the present findings.

The calcium and phosphate findings require similarly careful interpretation. In this cohort, mean serum calcium and phosphate were lower across worsening skeletal categories. This is compatible with the concept that bone health is influenced by the broader mineral environment, not merely by vitamin D status. Al-Khakani et al. reported differences in mineral parameters, including calcium and phosphorus, among postmenopausal women with osteoporosis [12]. In addition, magnesium and other micronutrients may influence bone metabolism, partly through interactions with vitamin D, calcium regulation, and parathyroid hormone pathways [13,14]. However, serum calcium and phosphate are regulated by multiple mechanisms, including parathyroid hormone, renal handling, diet, vitamin D status, and hormonal milieu. Since parathyroid hormone, magnesium, dietary intake, and renal-mineral regulatory markers were not included in the present analysis, the mineral findings should be viewed as supportive biochemical observations rather than mechanistic proof.

The present findings should also be interpreted in light of studies that have reported weak or nonsignificant associations between vitamin D and BMD. Tamimi et al. reported no significant correlation between 25(OH)D and BMD in patients undergoing peritoneal dialysis [15]. Triantafyllou et al. found no meaningful association between vitamin D levels and BMD in patients with multiple sclerosis [16]. Kamineni et al. also reported no correlation between serum 25(OH)D and BMD in a cohort of postmenopausal women, although vitamin D deficiency coexisted with low BMD [17]. Salamat et al. observed no significant relationship between several biochemical parameters and bone density in postmenopausal women with osteoporosis [18]. These apparently different findings are not necessarily contradictory. BMD is influenced by age, sex, menopausal status, body mass index, renal function, parathyroid hormone, medications, physical activity, nutrition, inflammatory status, and disease-specific factors. Variation in study population, skeletal site assessed, assay method, sample size, and adjustment for confounding variables may explain why vitamin D-BMD associations differ across studies.

One strength of the present study is that biochemical variables and DXA-defined skeletal categories were assessed together within the same hospital-based cohort. This provides a practical description of how serum 25(OH)D, calcium, phosphate, and BMD were distributed among adults undergoing bone health evaluation. The study also used standard vitamin D categories and standard densitometric definitions for normal BMD, osteopenia, and osteoporosis. These features support internal clarity of reporting.

Nevertheless, the limitations are important and should substantially temper interpretation. First, the cross-sectional design prevents assessment of temporality. It cannot determine whether lower vitamin D preceded bone loss or whether both were related to other unmeasured factors. Second, the sample was hospital-based and modest in size, limiting generalizability. Third, the equal allocation of 30 participants to each vitamin D category was useful for group-wise comparisons but does not reflect the natural prevalence of vitamin D sufficiency, insufficiency, or deficiency in the source population. Fourth, no formal a priori sample size calculation was performed, so the study should be considered exploratory rather than confirmatory. Fifth, the analysis was unadjusted. Important confounders, including parathyroid hormone, magnesium, body mass index, menopausal status, dietary calcium intake, sunlight exposure, physical activity, vitamin D supplementation history, bone turnover markers, and season of sample collection, were not available. Finally, detailed information on assay calibration, intra-assay and inter-assay variability, fasting status, and seasonal timing of vitamin D sampling was not available for analysis. These factors may have influenced the biochemical findings.

The clinical interpretation of this study should therefore be measured. The results are consistent with the established practice of interpreting vitamin D and mineral parameters as supportive biochemical information alongside DXA-based skeletal assessment. They do not suggest that serum 25(OH)D, calcium, or phosphate should replace densitometric evaluation, nor do they establish independent predictive value. Rather, the findings reinforce that vitamin D status and mineral profile may provide useful context when evaluating adults with low bone mass, provided that the results are interpreted in relation to clinical background and potential confounding factors.

## Conclusions

In this selected hospital-based cross-sectional cohort, participants with osteopenia and osteoporosis had lower mean serum 25(OH)D, BMD, calcium, and phosphate values than those with normal BMD. These findings are consistent with established links between vitamin D status, mineral metabolism, and skeletal health. However, the findings should be interpreted cautiously. The study was cross-sectional, modest in size, based on equal vitamin D group allocation, and limited by the absence of multivariable adjustment. Important determinants of bone metabolism, including parathyroid hormone, magnesium, menopausal status, body mass index, dietary intake, sunlight exposure, physical activity, and bone turnover markers, were not assessed. Therefore, the results should be considered descriptive associations rather than evidence of causality or independent prediction. Overall, the study supports the clinical value of interpreting serum 25(OH)D, calcium, and phosphate alongside DXA-based BMD assessment in adults undergoing bone health evaluation. Further adequately powered studies with broader metabolic profiling, representative sampling, and adjusted statistical analysis are required to clarify the independent contribution of vitamin D and mineral parameters to skeletal status.
